# Association between circadian physical activity trajectories and incident type 2 diabetes in the UK Biobank

**DOI:** 10.1038/s41598-024-57082-2

**Published:** 2024-03-18

**Authors:** Pufei Bai, Xian Shao, Lianqin Chen, Saijun Zhou, Yao Lin, Hongyan Liu, Pei Yu

**Affiliations:** 1 NHC Key Laboratory of Hormones and Development, Chu Hsien-I Memorial Hospital and Tianjin Institute of Endocrinology, Tianjin Medical University, No.6 North Huanrui Rd, Beichen District, Tianjin, China; 2 Tianjin Key Laboratory of Metabolic Diseases, Tianjin Medical University, Tianjin, 300134 China

**Keywords:** Diabetes, Risk factors, Lifestyle modification

## Abstract

Physical activity (PA) is linked to a decreased risk of type 2 diabetes mellitus (T2DM). However, the influence of circadian PA trajectories remains uncertain. This study aims to explore the optimal circadian PA trajectory pattern for reducing the risk of T2DM. Methods: A total of 502,400 participants were recruited from the UK Biobank between 2006 and 2010, and 102,323 participants provided valid accelerometer-captured acceleration data. After excluding individuals with prior T2DM, 99,532 participants were included in the final analysis. We initially investigated the association between PA intensity at 24 hourly time points and T2DM. Subsequently, PA trajectories were identified using K-means cluster analysis. Cox proportional hazard models were employed to estimate hazard ratios (HR). Four distinct PA trajectories were identified: consistently low, single peak, double peak, and intense trajectories. Compared to consistently low, single peak, double peak and intense PA trajectory reduced the risk of T2DM progressively. Sensitivity analyses, further excluding individuals with glycated hemoglobin (HbA1c) ≥ 6.5% or random glucose ≥ 11.1 mmol/L and adjusted for daily average acceleration, yielded consistent results. This confirms that the ideal circadian PA trajectory serves as a protective factor, independently of PA intensity. Subgroup analyses indicated that these effects were more pronounced in men and individuals with eGFR < 60 mL/(min*1.73 m^2^). In conclusion, ideal circadian PA trajectory patterns (especially intense and then double peak) reduced risk of T2DM.

## Introduction

Physical activity (PA) is a preventive factor for type 2 diabetes mellitus (T2DM)^1–3^, yet the role of circadian PA trajectory patterns remains relatively unexplored. Most studies, such as those by Duncan^4^ and Castetbon^5^, rely on questionnaires to gauge PA metrics. These questionnaires are often crude and subjective, overlooking many nuances of PA. Additionally, an increasing of research has delved into the relationship between circadian rhythm and health outcomes^6–10^. Disruptions in circadian rhythm have been associated with a range of diseases, including obesity^11^, cardiovascular events^7^, and mortality^12^. Growing evidence also suggests that the pattern of PA is an independent determinant of T2DM risk^11^. However, recommendations regarding the optimal PA patterns remain under discussion. Given the challenges of real-time activity data collection, it remains unclear which PA pattern offers the greatest health benefits, especially in the absence of evidence from large-scale cohorts. Few studies have addressed the relationship between PA trajectories and type 2 diabetes. The advent of wrist-worn triaxial accelerometers^13^ has bridged this gap, capturing continuous activity data in a precise manner, facilitating research into the optimal 24-h PA trajectory patterns. Furthermore, cluster trajectory models offer a more intuitive, granular approach to identify potential subgroups based on temporal measures, while trajectory models are traditionally suited for cohort data^14–16^, the repeated measures of PA acceleration captured by accelerometer bear similarities. Previous studies often singularly focused on either the intensity or the variation of PA, which was somewhat one-sided. In this study, through K-means clustering trajectory analysis, we identified the PA trajectories in the population that encompass both aspects. This provides a more comprehensive and precise depiction, capturing the actual PA behaviours and patterns of individuals. Thus, trajectories of circadian PA patterns might offer fresh insights.

Therefore, our study, based on a large population cohort from the UK Biobank, identifies circadian PA trajectories. We aimed to evaluate the optimal PA trajectories by examining their association with type 2 diabetes incidence. We further sought to reinforce the robustness of our findings through sensitivity analyses and to uncover variations in specific populations via subgroup analyses.

## Results

### Identification of circadian PA trajectories

**Figure 1:**
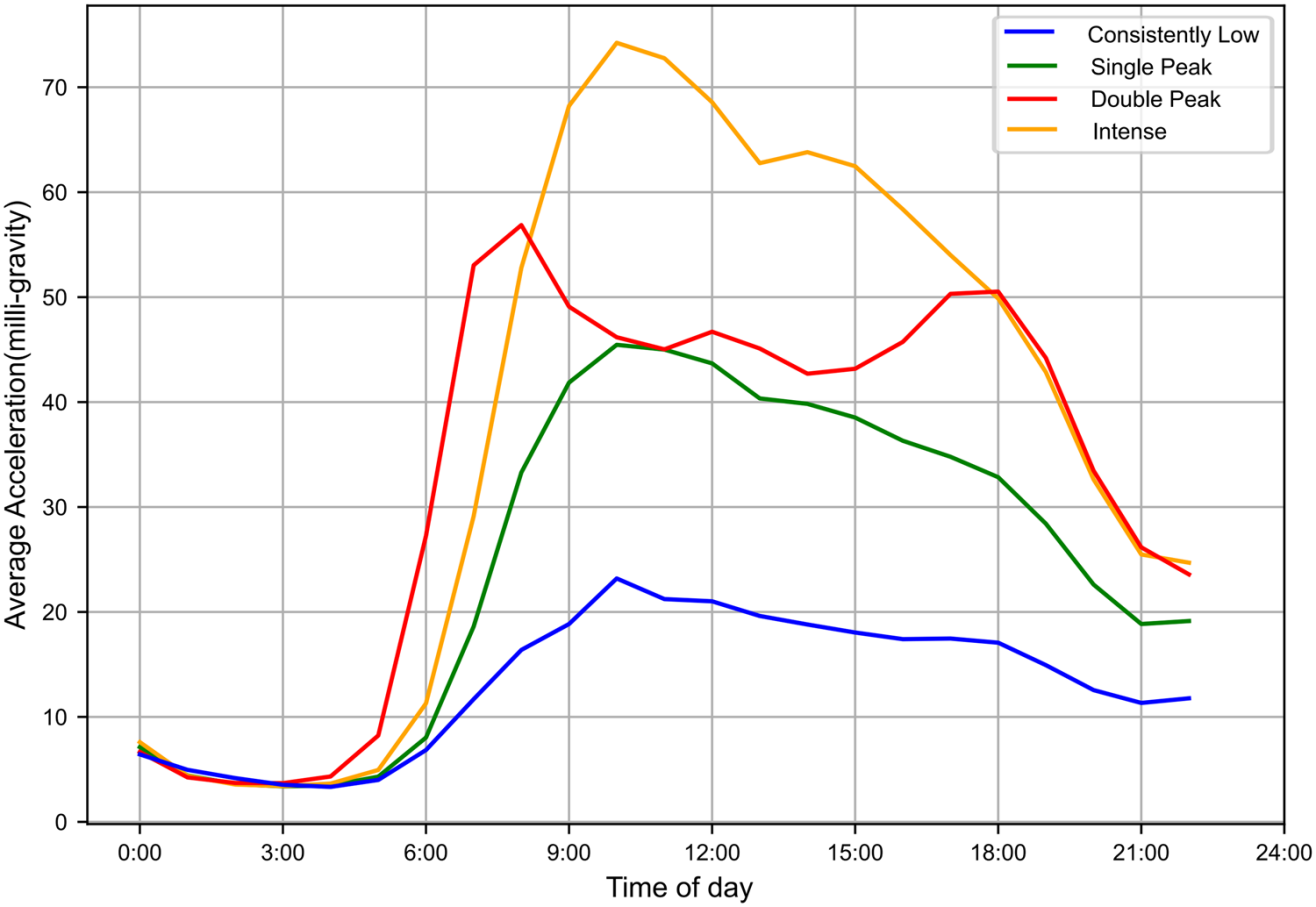
Trajectory groups of PA patterns in this study. Trajectory Cluster were based on continuous monitoring acceleration data and circadian rhythm temporal distribution from accelerometer over a span of 24 h.

Based on continuous monitoring acceleration data and circadian rhythm temporal distribution from accelerometer over a span of 24 h of participants in this study, the K-means clustering algorithm discerned four potential trajectory groups, as depicted in Fig. 1. The first group, termed the “consistently low Group”, comprised 13.8% of the participants. This group demonstrated consistently lower daily PA acceleration levels throughout the day, below 23.37 mg (milligravity). The second group, encompassing 47.7% of the participants, was termed “single peak”. This group was characterized by relatively higher PA acceleration levels in the morning, reaching 45.54 mg. In contrast, the third group, termed “double peak”, consisted of 18.8% of participants. This group displayed two distinct PA level peaks: one in the morning at 57.09 mg and another later in the afternoon at 50.65 mg. The fourth group, termed “intense”, accounted for 19.7% of participants, characterized by high PA acceleration levels during the day, peaking at a 74.26 mg.

### Participant characteristics across different trajectory groups

Our analysis encompassed 99,532 participants, with 42,902 men and 56,630 women. The mean age at baseline was 56 years, standard deviation (SD) 7.8 years. 13.8% of participants (Group 1) displayed a relatively steady low level of PA during the day, without evident PA intensity peaks.

Typically, these participants were older men with a higher BMI and smoking history. In contrast, 19.7% of participants (Group 4) exhibited higher activity intensities. This group predominantly consisted of younger women with higher education and income levels. Detailed demographic and feature breakdowns are provided in Table 1; Table S1.

**Table 1:** Characteristics of participants in the four PA trajectory groups in the UK Biobank Study.

Trajectory cluster number	Overall	Consistently low	Single peak	Double peak	Intense	p
N (%)	99,532	13,764(13.8%)	47,500(47.7%)	18,681(18.8%)	19,587(19.7%)	
Men (%)	42,902 (43.1)	7096 (51.6)	20,350 (42.8)	7693 (41.2)	7763 (39.6)	< 0.001
Age	56.0 (7.8)	57.3 (7.9)	57.5 (7.4)	51.9 (7.6)	55.3 (7.5)	< 0.001
Race and ethnicity (%)						< 0.001
White	96,444 (96.9)	13,263 (96.4)	46,303 (97.5)	17,761 (95.1)	19,117 (97.6)	< 0.001
Mixed	550 (0.6)	78 (0.6)	210 (0.4)	166 (0.9)	96 (0.5)	< 0.001
Asian or Asian British	1160 (1.2)	215 (1.6)	461 (1.0)	322 (1.7)	162 (0.8)	< 0.001
Black or Black British	844 (0.8)	131 (1.0)	313 (0.7)	289 (1.5)	111 (0.6)	< 0.001
Other ethnic group	534 (0.5)	77 (0.6)	213 (0.4)	143 (0.8)	101 (0.5)	< 0.001
Educational level (%)						< 0.001
College or University degree	43,569 (43.8)	5778 (42.0)	20,446 (43.0)	9188 (49.2)	8157 (41.6)	< 0.001
A levels/AS levels or equivalent	13,159 (13.2)	1805 (13.1)	6170 (13.0)	2623 (14.0)	2561 (13.1)	< 0.001
O levels/GCSEs or equivalent	20,379 (20.5)	2746 (20.0)	9836 (20.7)	3590 (19.2)	4207 (21.5)	< 0.001
CSEs or equivalent	4038 (4.1)	510 (3.7)	1701 (3.6)	821 (4.4)	1006 (5.1)	< 0.001
NVQ or HND or HNC or equivalent	5293 (5.3)	862 (6.3)	2578 (5.4)	820 (4.4)	1033 (5.3)	< 0.001
Other	13,094 (13.2)	2063 (15.0)	6769 (14.3)	1639 (8.8)	2623 (13.4)	< 0.001
Household income (%)						< 0.001
Low	14,457 (14.5)	2588 (18.8)	7497 (15.8)	1706 (9.1)	2666 (13.6)	< 0.001
Medium	53,373 (53.6)	7287 (52.9)	26,211 (55.2)	9193 (49.2)	10,682 (54.5)	< 0.001
High	31,702 (31.9)	3889 (28.3)	13,792 (29.0)	7782 (41.7)	6239 (31.9)	< 0.001
BMI, kg/m^2^	26.6 (4.4)	28.4 (5.2)	27.0 (4.4)	25.9 (4.1)	25.3 (3.6)	< 0.001
Smoking status (%)						< 0.001
Never	57,116 (57.4)	7189 (52.2)	26,964 (56.8)	11,458 (61.3)	11,505 (58.7)	< 0.001
Previous	35,445 (35.6)	5077 (36.9)	17,292 (36.4)	6076 (32.5)	7000 (35.7)	< 0.001
Current	6971 (7.0)	1498 (10.9)	3244 (6.8)	1147 (6.1)	1082 (5.5)	< 0.001
eGFR, mL/(min*1.73 m^2^)	91.3 (12.6)	89.6 (13.7)	90.0 (12.6)	94.6 (12.1)	92.6 (11.9)	< 0.001
CRP, mg/L	2.2 (3.9)	3.0 (4.9)	2.4 (3.9)	1.9 (3.5)	1.8 (3.3)	< 0.001
Medical history						
Cardiovascular disease (%)	11,937 (12.0)	2307 (16.8)	6176 (13.0)	1556 (8.3)	1898 (9.7)	< 0.001
Cancer (%)	10,617 (10.7)	1610 (11.7)	5402 (11.4)	1671 (8.9)	1934 (9.9)	< 0.001

Asian or Asian British: Individuals of Asian descent, either born in the UK or as immigrants. Black or Black British: individuals of African or Caribbean descent, either born in the UK or as immigrants. College or University Degree: Completion of college or university level education. A levels/AS levels or equivalent: Completion of education comparable to advanced high school. O levels/GCSEs or equivalent: Completion of general secondary education, comparable to middle school education. CSEs or equivalent: Completion of a basic education level, considered more foundational. NVQ or HND or HNC or equivalent: vocational and professional education, focusing on practical skills and job training.

### Hourly PA and incident type 2 diabetes

**Figure 2:**
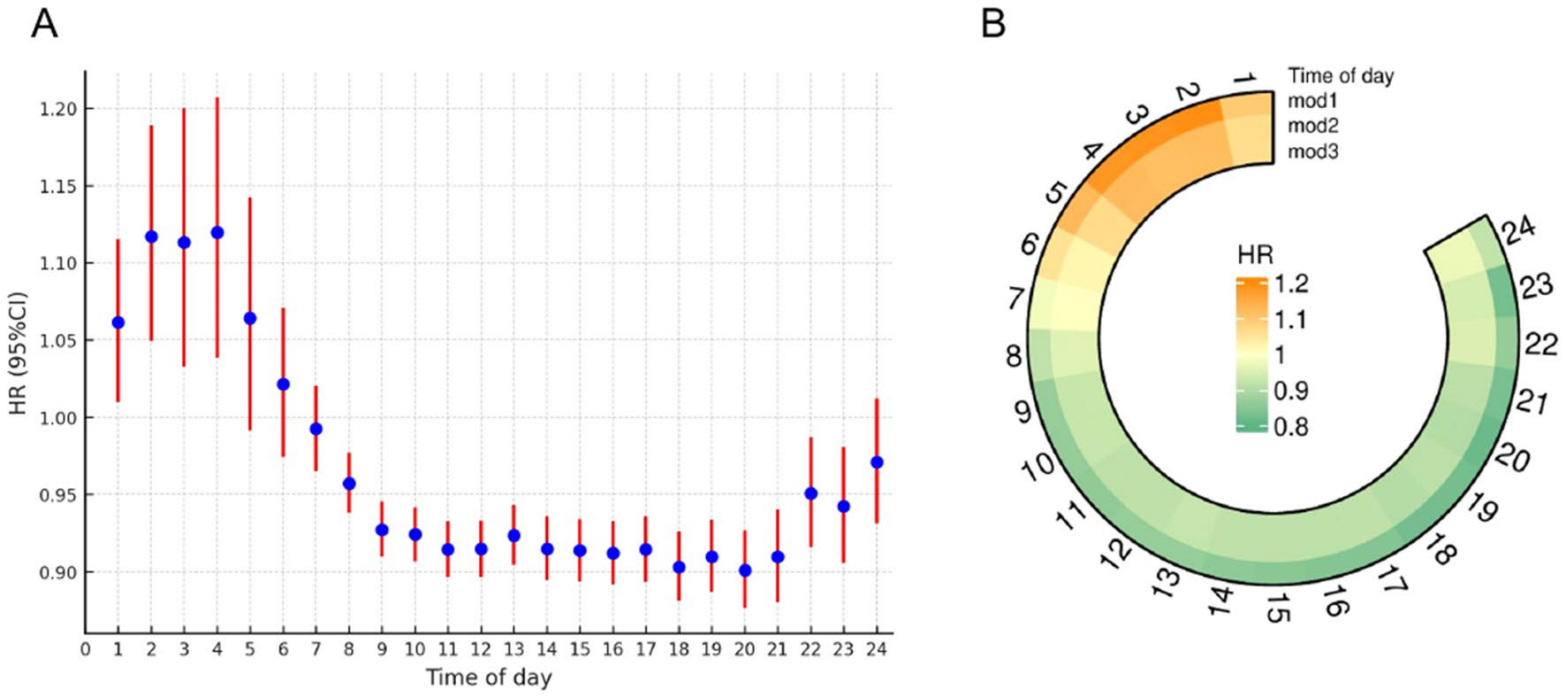
Hourly PA and T2DM risk distribution in 24 h. (**A**) shows the risk on incident T2DM risk in mod3; (**B**) also shows association of T2DM risk with 24-hourly acceleration in mod1 and mod2. Model 1 adjusted for gender, age, and ethnicity. Model 2 further adjusted for education level, income, BMI, and smoking based on Model 1. Model 3 further adjusted for eGFR, CRP, and medical history of cardiovascular disease and cancer based on Model 2.

During the follow-up period, 2,692 incident diabetes cases were identified according to ICD-10 codes: E11. Association between PA over 24 h and T2DM were evaluated, higher relative PA during nighttime hours (00:00–6:00am) was associated with increased risk, while morning (8:00–11:00am) and late afternoon (18:00–20:00pm) physical activities were linked to lower risk (Fig. 2A, in mod3 fully adjusted for gender, age, ethnicity, education level, income, BMI, smoking status, eGFR, CRP, and medical history of cardiovascular disease and cancer). The highest HR (95% CI) for T2DM with Hourly PA occurred at 4:00 am, with a value of 1.12 (1.04–1.21), while the two lowest HR (95% CI) values for T2DM were observed at 20:00 pm, with a value of 0.90 (0.88–0.93), and at 11:00 am, with a value of 0.91 (0.90–0.93).

Figure 2B shows HR (95% CI) for T2DM with hourly PA in different models. And the HR (95% CI%) corresponding to specific 24-hourly PA are presented in Table S2, as well as the outcomes for the population further excluded individuals with HbA1c levels ≥ 48 mmol/mol (6.5%) or random glucose levels ≥ 11.1 mmol/L at baseline according to ADA standards presented in Figure S1, Table S3. This preliminary analysis suggests an association between the time distribution of PA throughout the day and T2DM risk.

### Association between PA trajectory groups and T2DM incidence

**Figure 3:**
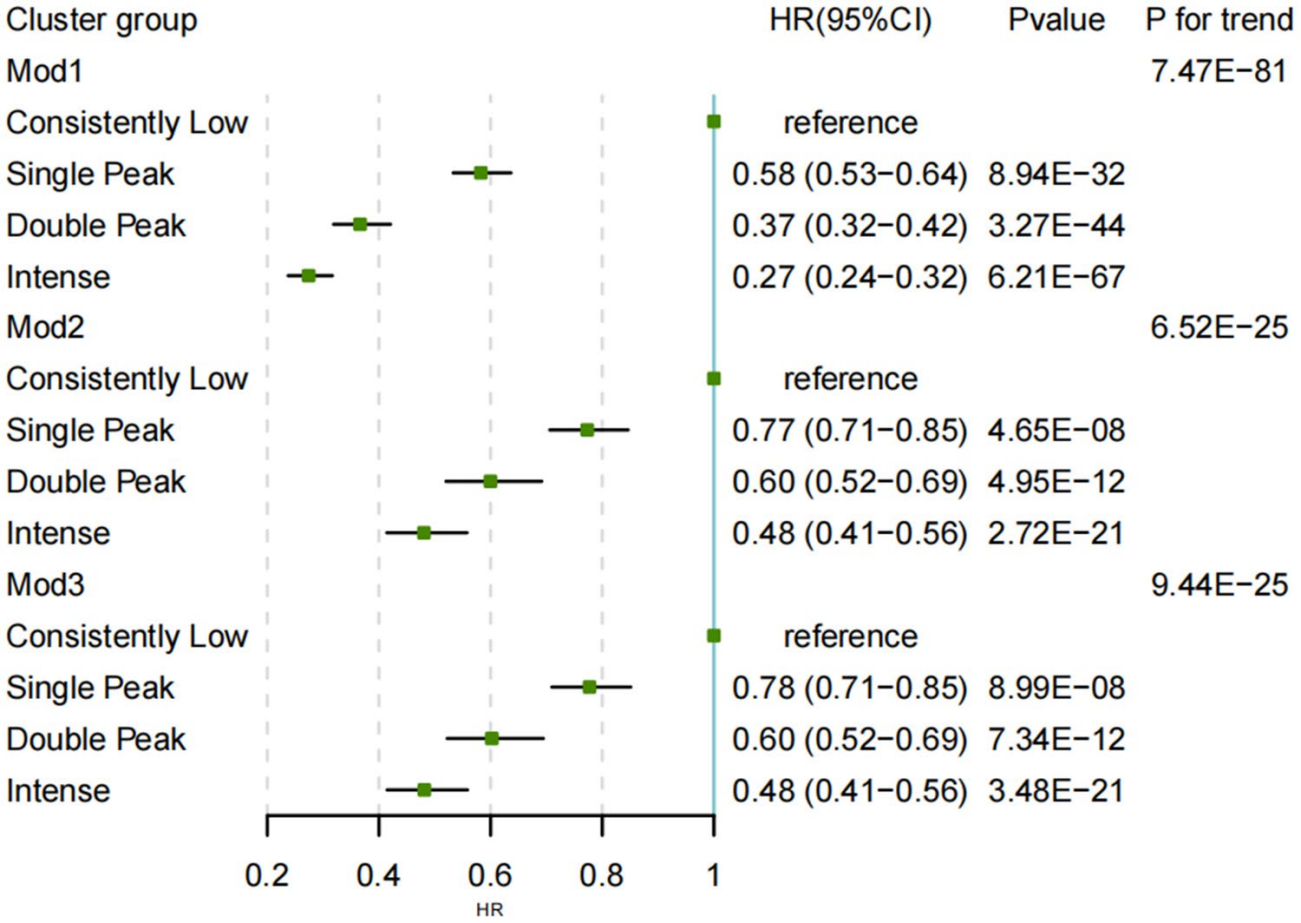
Association of incident T2DM with PA trajectories in mod1, mod2 and mod3. Model 1 adjusted for gender, age, and ethnicity. Model 2 further adjusted for education level, income, BMI, and smoking based on Model 1. Model 3 further adjusted for eGFR, CRP, and medical history of cardiovascular disease and cancer based on Model 2.

Based on PA trajectories identified by continuous PA trends, Fig. 3 provided a visual representation for the associations between circadian PA trajectories and T2DM. Compared to the consistently low group, the single peak, double peak, and intense groups displayed progressively reduced risks associated with overall T2DM incidence. Specifically, in comparison to the consistently low pattern, the HR (95% CI) of T2DM risk with PA trajectories in single peak, double peak, and intense trajectories were 0.78 (0.71–0.85), 0.60 (0.52–0.69), and 0.48 (0.41–0.56) respectively in mod3, fully adjusted for gender, age, ethnicity, education level, income, BMI, smoking status, eGFR, CRP, and medical history. In mod1 and mod2, ideal circadian exercise trajectory patterns similarly displayed an inverse association with T2DM risk, the HR (95% CI) in single peak, double peak, and intense trajectories were respectively 0.58(0.53–0.64), 0.37(0.32–0.42), 0.27 (0.24–0.32) and 0.77 (0.71–0.85), 0.60 (0.52–0.69), 0.48 (0.41–0.56). Association of incident T2DM with 24-hourly PA acceleration using the single peak, double peak, and intense group as a reference, yielded consistent results (Figure S2).

### Sensitivity analysis

To ensure test result stability, further exclusions were made from those diagnosed with T2DM, and individuals with random glucose levels ≥ 11.1 mmol/L or HbA1c levels ≥ 48 mmol/mol (6.5%). The associations between PA trajectory groups and T2DM incidence remained consistent (Figure S3). Compared to the consistently low group, the HR (95% CI) of T2DM risk with PA trajectories for single peak, double peak, and intense trajectories were 0.60 (0.54–0.66), 0.36 (0.31–0.42) and 0.28 (0.24–0.33) in mod1, 0.79 (0.71–0.87) 0.59 (0.50–0.69) and 0.49 (0.42–0.58) in mod2, 0.80 (0.72–0.89),0.61 (0.52–0.73) and 0.50 (0.42–0.60) in mod3.

Additionally, we further adjusted for all-day average acceleration on top of all confounders in mod3. The ideal PA trajectory groups remained a significant protective factor for T2DM, compared to the consistently low group, the HR (95% CI) for single peak, double peak, and intense trajectories were 0.90 (0.80–1.02), 0.79 (0.64–0.96) and 0.68 (0.54–0.86) (Figure S4). Besides, considering some relevant occupational factors, we adjusted for jobs involving night shifts, PA work and unemployment, and yielded consistent results (Figure S5).

### Subgroup analysis

We conducted subgroup analyses based on gender, age, BMI, eGFR, CRP, and medical history of cardiovascular disease and cancer. As showed in Table 2, the protective trend of ideal exercise trajectories remained consistent across all subgroups. However, the strength of the protective effect varied, especially for men and those with eGFR < 60mL/(min*1.73 m^2^). HR (95% CI) of T2DM risk with PA trajectories for single peak, double peak, and intense trajectories were 0.76 (0.65–0.88), 0.70 (0.56–0.88) and 0.54 (0.43–0.69) in women; 0.80 (0.71–0.89), 0.54 (0.45–0.65) and 0.44 (0.37–0.54) in men; 0.78 (0.71–0.86), 0.61 (0.53–0.71) and 0.49 (0.42–0.57) in population with eGFR ≥ 60 mL/(min*1.73 m^2^); 0.71 (0.44–1.14), 0.33 (0.10–1.13) and 0.26 (0.08–0.86) in population with eGFR < 60 mL/(min*1.73 m^2^). Double peak and intense PA trajectories offered more pronounced protection subgroups of men and eGFR < 60mL/(min*1.73 m^2^). Specifically, compared to the consistently low pattern, the T2DM risk reduction for double peak, and intense groups were 30% and 46% for women; 46% and 56% for men; 39% and 51% for eGFR ≥ 60 mL/(min*1.73 m^2^); 67% and 74% for eGFR < 60 mL/(min*1.73 m^2^).

**Table 2:** Stratified analyses for association between PA trajectory group and incident T2DM.

Subgroups	PA trajectory group				*P* for trend
	Consistently low	Single peak	Double peak	Intense	
Age					
<65	1 (reference)	0.80 (0.71–0.89)	0.61 (0.52–0.72)	0.49 (0.41–0.58)	2.43E-19
≥ 65	1 (reference)	0.74 (0.63–0.88)	0.59 (0.41–0.84)	0.49 (0.35–0.68)	1.52E-06
Sex					
Men	1 (reference)	0.80 (0.71–0.89)	0.54 (0.45–0.65)	0.44 (0.37–0.54)	8.76E-20
Women	1 (reference)	0.76 (0.65–0.88)	0.70 (0.56–0.88)	0.54 (0.43–0.69)	9.55E-07
BMI					
< 25	1 (reference)	0.66 (0.48–0.90)	0.63 (0.42–0.93)	0.52 (0.36–0.75)	3.42E-03
≥ 25	1 (reference)	0.78 (0.71–0.85)	0.59 (0.51–0.69)	0.47 (0.40–0.56)	2.46E-22
eGFR					
< 60	1 (reference)	0.71 (0.44–1.14)	0.33 (0.10–1.13)	0.26 (0.08–0.86)	7.05E-03
≥ 60	1 (reference)	0.78 (0.71–0.86)	0.61 (0.53–0.71)	0.49 (0.42–0.57)	2.61E-23
CRP					
< Median	1 (reference)	0.64 (0.54–0.77)	0.48 (0.37–0.63)	0.41 (0.32–0.54)	7.44E-12
≥ Median	1 (reference)	0.82 (0.74–0.92)	0.68 (0.57–0.80)	0.54 (0.45–0.64)	9.77E-13
CVD history					
No	1 (reference)	0.75 (0.68–0.83)	0.57 (0.49–0.67)	0.47 (0.40–0.55)	8.19E-22
Yes	1 (reference)	0.84 (0.70–1.01)	0.73 (0.53–1.01)	0.51 (0.36–0.72)	1.31E-04
Cancer history					
No	1 (reference)	0.77 (0.69–0.84)	0.59 (0.51–0.68)	0.48 (0.41–0.56)	9.25E-23
Yes	1 (reference)	0.87 (0.67–1.14)	0.73 (0.48–1.11)	0.48 (0.30–0.78)	2.43E-03

## Discussion

In this large-scale prospective cohort study based on the UK Biobank, we identified four distinct PA trajectory patterns according to intensity and circadian rhythm distribution. We discovered that the optimal PA trajectory offers protective effects against the onset of T2DM. PA remains one of the most evident cornerstones in the prevention of T2DM. Our study adds fresh insights to previous evidence, suggesting that the PA trajectory is another independent influencer of T2DM risk, introducing a new dimension to T2DM risk prevention.

To our knowledge, this is the first study to prospectively analyse the relationship between 24-h PA trajectory patterns and T2DM incidence in a large population. By continuously monitoring the activity of nearly 100,000 individuals over seven days using accelerometer, this study allowed for the exploration of circadian rhythm distribution and the cumulative effects of PA. We discerned four PA trajectories: consistently low, single peak, double peak, and intense group. These results further elucidate the relationship between PA trajectories patterns and health outcomes.

In our analysis, older men exhibited relatively lower PA levels. In contrast, younger, highly-educated women showed a propensity for more intense PA or increased activity in the late afternoon. Numerous studies indicate that PA patterns are influenced by various factors, including physiological, physical environment, social background, and occupational status^5,13^. For older participants, their daytime PA pattern peaks tend to be lower, consistent with previous findings^17^. On the other hand, younger individuals have higher daily activity levels and tend to engage in PA later in the day, likely due to indoor occupations commonly held by younger cohorts^18,19^. Clearly, various PA patterns exist within populations. Analysing these patterns using accelerometer can further identify target populations in need of exercise interventions, offering insights potentially beneficial to public health policy strategies.

Importantly, our study observed variations in T2DM incidence across the four identified PA trajectory groups. After adjusting for all confounders, optimal PA trajectory patterns progressively revealed protective effects against T2DM onset, especially evident in the intense and then double peak trajectory patterns. While consistently low group had the highest T2DM incidence, with PA levels below the World Health Organization's recommendations^20^, in line with previous findings linking physical inactivity to increased T2DM risk^21^. Conversely, the "intense group" had the lowest risk. While past studies have shown that any PA offers some benefits over sedentary behaviors^21–23^, our findings underscore the need for exercise to attain specific intensities to maximize health benefits against T2DM onset.

Crucially, even when reaching similar PA intensity peaks, the protective effects of different circadian rhythms differ. We observed that the double peak and single peak groups had comparable peak intensities. However, double peak group, engaging in some activity later in the afternoon, seems to correlate with stronger protective effects, consistent with past studies suggesting that afternoon physical activities have a more positive impact on health^24^. Although some studies have shown associations between morning PA and better metabolic health^25–27^, others have found that afternoon exercises more effectively improved blood sugar levels than morning exercises^28,29^. Furthermore, afternoon exercises were more effective in elevating blood lipid levels than morning exercises^24,30,31^, consistent with the significant circadian rhythm characteristics of cholesterol synthesis, with its lowest during the day and peaking at night^32,33^. In combination with our study's findings, this underscores the significance of the double peak exercise pattern in preventing T2DM. These findings emphasize that even if some patients face difficulties in maintaining continuous intense exercises, engaging in intermittent moderate-intensity activities throughout the morning and afternoon still provides health benefits.

Sensitivity analyses, further excluding populations according to ADA standards and further adjusting for all-day average acceleration, yielded consistent results, proving that ideal PA trajectory are protective factors against type 2 diabetes, independent from the overall daily average PA intensity. These insights are immensely valuable for clinical practice, highlighting the comprehensive effects of an ideal PA trajectory of all-day exercise regimen on health.

In subgroup analyses based on gender, age, BMI, eGFR, CRP, and medical history of cardiovascular disease and cancer, we observed that the ideal circadian exercise trajectory pattern consistently showcased a protective trend against T2DM risk. Especially in men, the double peak and intense patterns offered stronger protection, possibly due to men generally having more adverse lifestyle habits like higher BMI, smoking, etc^34^, while the PA trajectory pattern may play a role in improving some of these factors, such as BMI, and they are yet to be further studied as potential mediators affecting the occurrence of T2DM. Simultaneously, the ideal circadian exercise trajectory pattern provided stronger protection for eGFR < 60mL/(min*1.73 m^2^), possibly because individuals with impaired kidney function have reduced metabolic capabilities^35–37^. The ideal circadian exercise trajectory pattern might regulate the state of circadian metabolic disorder, subsequently reducing diabetes risk. This further emphasizes that in specific populations, the individualized and rational design of exercise patterns, considering exercise intensity and rhythmicity, can help achieve greater health benefits and investigation is needed to explore the underlying mechanisms in greater depth.

This study has several strengths, including its vast sample size and a well-characterized cohort from the UK Biobank. Moreover, PA in the UK Biobank was objectively collected by e wrist-worn accelerometers which can be used to estimate population levels of AEE and TEE in free-living conditions with high precision^38^ and explain about 44–47% of the variation in PA energy expenditure^39^. We further employed a data-driven clustering method to identify subgroups of the circadian PA trajectory pattern, more likely representing our participants' natural behavioural pattern than previously used predefined time periods. Lastly, we had the capability to examine the association between circadian PA trajectory patterns and T2DM incidence due to our extensive medical records and baseline data.

We recognize certain limitations in our study. Although we made rigorous efforts to adjust for confounders, the possibility of other unconsidered confounding factors remains. Secondly, given our study's primary focus on a European population cohort, the generalizability of our findings may require validation on a global scale. In this study, through K-means cluster analysis of accelerometer-related data provided by the UK Biobank dataset, four trajectory groups were identified. However, due to variations in economic status, regional culture, and climatic conditions, we acknowledge the potential existence of different PA patterns in other populations, such as an "Evening Only" pattern. While the result of association of hourly PA with T2DM showed that those with higher PA during nighttime hours had a higher risk of T2DM. However, the current study data suggested that, in this British cohort, the proportion of individuals with such a pattern was too small to be identified as a separate cluster. Therefore, future research could consider exploring other potential PA patterns in different regional populations and further investigate their impact on health outcomes, particularly focusing on nighttime PA.

Despite these limitations, this study, with ts strengths, offers fresh content to an unexplored research area, discussing the ideal circadian PA trajectory pattern for preventing T2DM. This lays the groundwork for devising rational physical exercise strategies. If replicated and validated in broader populations in the future, it stands to benefit public health immensely.

## Conclusion

Optimal PA trajectories reduced T2DM incidence. The protective effect was not just linked to intensity but also with circadian rhythm distribution. This might suggest that an ideal circadian PA intensity distribution could be an additional beneficial behavioural factor to maximize health benefits and reduce T2DM risk.

## Methods

### Study design and population

This study utilized data collected from the UK Biobank, a large-scale prospective study that recruited 502,400 adult participants between 2006 and 2010. The study amassed a wealth of demographic data and laboratory measurements while continually monitoring various health-related outcomes. Researchers can access de-identified data after signing a material transfer agreement, committing to use it solely for approved research and not attempting to identify any participants. After the exclusion of participants without accelerometer data, a total of 102,323 individuals were considered. Further excluding those with a prior history of T2DM, individuals (n = 99,532) without T2DM at baseline were incorporated into the final T2DM incidence risk analysis (Figure S6).

The present study protocol adhered to the ethical guidelines of the 1975 Declaration of Helsinki and approved by the ethics committee of Tianjin Chu Hsien-l Memorial Hospital. The study granted access to the UK Biobank database, which received ethical approval from the North West Multi-Centre Research Ethics Committee (21/NW/0157). All participants provided informed consent.

### Accelerometer data and PA trajectories

To objectively measure PA, participants wore the Axivity AX3 wrist-worn triaxial accelerometer, capturing acceleration data continuously over 7 days at a frequency of 100Hz with a dynamic range of ± 8g. Participants wore the device uninterrupted, engaging in their typical activities. During a period of seven days, 24-h acceleration measurements provided real-time monitoring data that helped assess PA. Data processing was carried out by the UK Biobank (UKB) expert working group. The details of data processing were elaborately described in a previously published article^40^. The preliminary processed acceleration data, which was a movement metric (Euclidean norm minus one, ENMO) measured in milligravity (mg), served as a summary indicator of the body's acceleration (Figure S7). This metric reflected the instantaneous intensity of PA and was directly provided by the official UK Biobank, which constituted the raw data accessible for analysis in this study.

### Evaluation of Type 2 diabetes

Type 2 diabetes was classified based on International Statistical Classification of Diseases, Tenth Revision (ICD-10) codes: E11. Also, according to the American Diabetes Association (ADA) criteria^41^, those with baseline glycated hemoglobin (HbA1c) levels ≥ 48 mmol/mol (6.5%) or random glucose levels ≥ 11.1 mmol/L were further excluded for sensitivity analysis. Follow-up for T2DM incidence continued until February 1, 2022.

### Covariates

Factors such as age, gender, ethnicity, household income, smoking status, BMI, eGFR, CRP, and medical history were collected. Additional details regarding covariate measurements can be found in the UK Biobank online protocol (https://www.ukbiobank.ac.uk).

### Statistical analysis

In order to identify potential PA trajectories, we employed the K-means algorithm by “sklearn.cluster” package in Python to perform cluster analysis using the average acceleration values for each of the 24-h periods across all measurement days, provided officially by the UK Biobank. Before conducting the cluster analysis, a Within-Cluster Sum of Squares (WSS) plot was created to determine the number of clusters to be considered in the K-means analysis (Figure S8).

All other analyses were conducted in R 4.2.2. Given the valuable nature of the continuous 24-h exercise data, multivariate imputation of the random forest method by “mice” package was used and only a small percentage of missing values for some covariates were imputed, maximizing data retention The distribution before and after imputation remained consistent, ensuring result reliability (see Table S4). Baseline demographic differences between the four trajectory groups (Table 1) were analysed. For normally distributed continuous variables, ANOVA was applied, for inappropriate distributions and categorical variable, the Kruskal–Wallis test and chi–square test were employed. Cox Proportional Hazards model was conducted by “survival” package. Proportional hazard assumptions were checked using Schoenfeld residuals and visually (Figure S9). Based on the identified trajectories from cluster analysis, we evaluate the association between PA patterns and T2DM risk. Three models were fitted to assess the effect of covariates. Model 1 adjusted for gender, age, and ethnicity. Model 2 further adjusted for education level, income, BMI, and smoking based on Model 1. Model 3 further adjusted for eGFR, CRP, and medical history of cardiovascular disease and cancer based on Model 2.

To ascertain result stability, sensitivity analyses were conducted: (1) further excluding individuals with HbA1c levels ≥ 48 mmol/mol (6.5%) or random glucose levels ≥ 11.1 mmol/L at baseline; (2) adopting Model 4, which adjusted for all-day average acceleration on top of all confounders adjusted in Model 3. Additionally, subgroup analyses were conducted based on gender, age, BMI, eGFR, CRP, and medical history of cardiovascular disease and cancer. Adequate analysis was performed to ensure the stability of the results. However, to address the issue of multiple testing, the Benjamini and Hochberg method was used to adjust the p-values and p-values after adjustment < 0.05 were considered significant.

### Ethics approval and consent to participate

The study protocol adhered to the ethical guidelines of the 1975 Declaration of Helsinki and the UK Biobank obtained ethics approval from the North West Multi-Centre Research Ethics Committee (21/NW/0157). Informed consent was obtained from all individual participants included in the study.

### Supplementary Information

Supplementary Information.

## Data Availability

The data are available from the UK Biobank, but there are restrictions on their availability. These data were used under a license for the current study and are not publicly accessible. Researchers who wish to access the UK Biobank database will need to apply for access through the following link: https://www.ukbiobank.ac.uk/enable‑your‑research/.
