# Supplementary material for: Association between circadian physical activity trajectories and incident type 2 diabetes in the UK Biobank
Source: Sci Rep. 2024 Mar 18;14:6459. doi: 10.1038/s41598-024-57082-2 (PMC10948909; doi:10.1038/s41598-024-57082-2)
Supplement: Supplementary file 1 — Supplementary Information. [file 41598_2024_57082_MOESM1_ESM.docx]

Supplementary Material

## Supplementary Figures

## Figure S1. Association of incident T2DM with 24-hourly physical activity acceleration, further excluding individuals with HbA1c levels≥48 mmol/mol (6.5%) or random glucose levels≥11.1 mmol/L at baseline according to ADA standards.

##
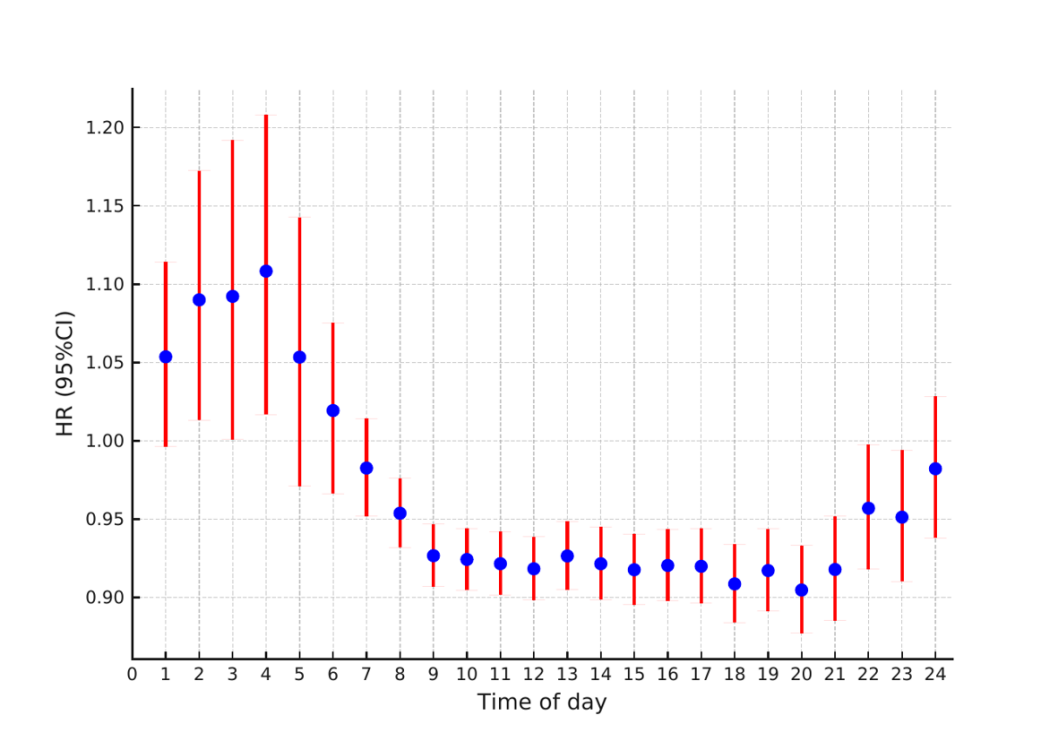


**Figure S2.** Association of incident T2DM with 24-hourly physical activity acceleration, using the a) single peak, b) double peak, and c) intense group as a reference.
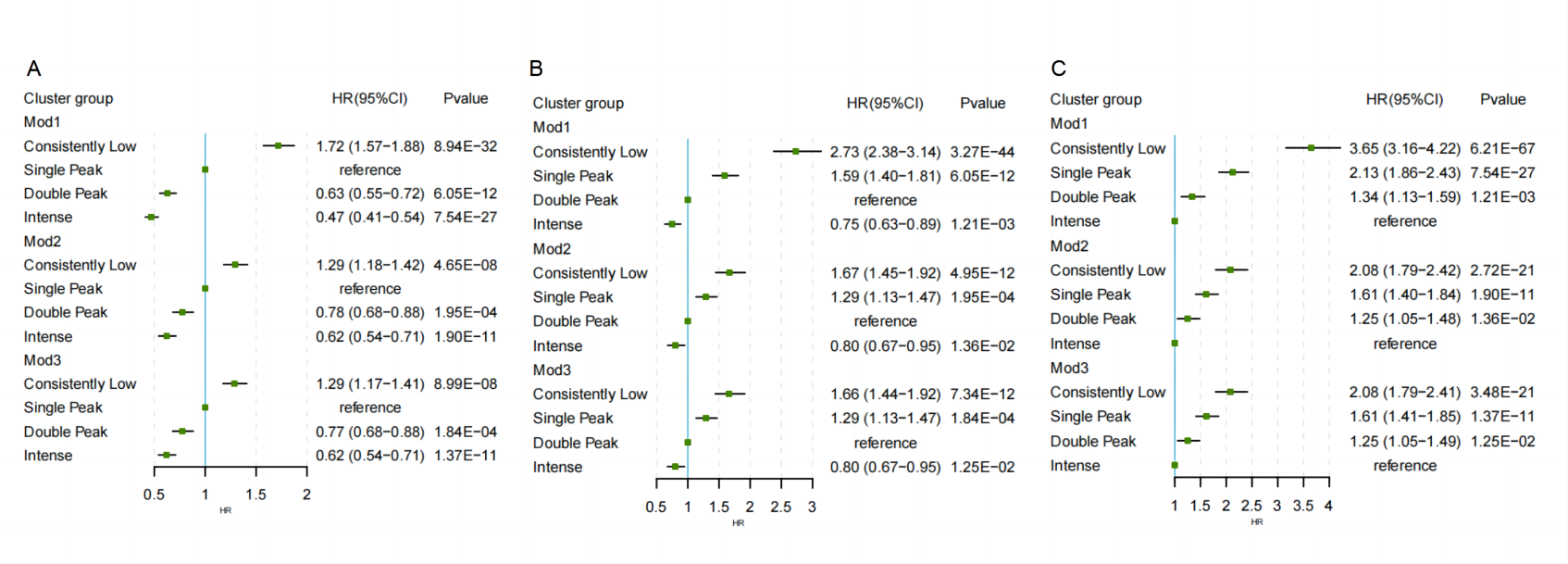


**Figure S3.** Association of incident T2DM with physical activity trajectories, further excluding individuals with HbA1c levels≥48 mmol/mol (6.5%) or random glucose levels≥11.1 mmol/L at baseline.


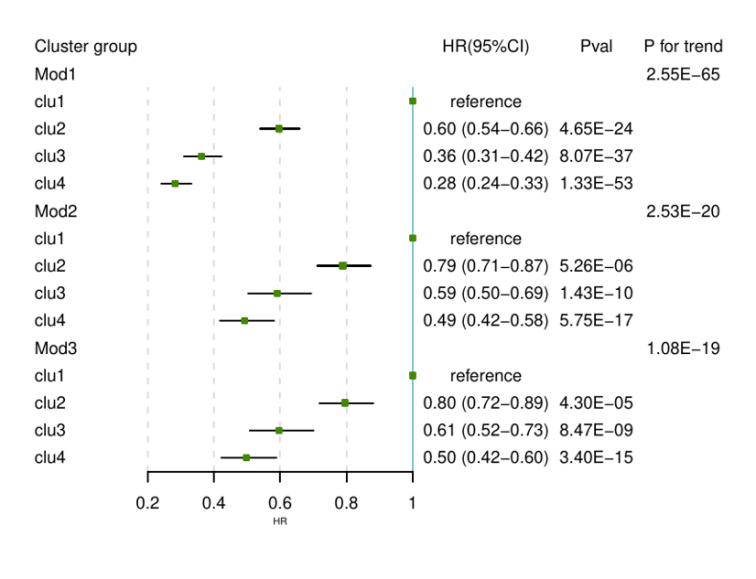


**Figure S4.** Association of incident T2DM with physical activity trajectories, further adjusted all-day average acceleration based on mod3.


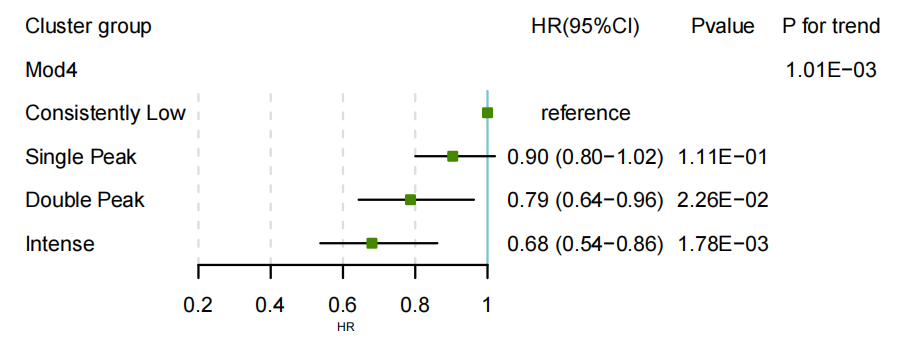


**Figure S5.** Association of incident T2DM with physical activity trajectories, further adjusted related job factors based on mod3.


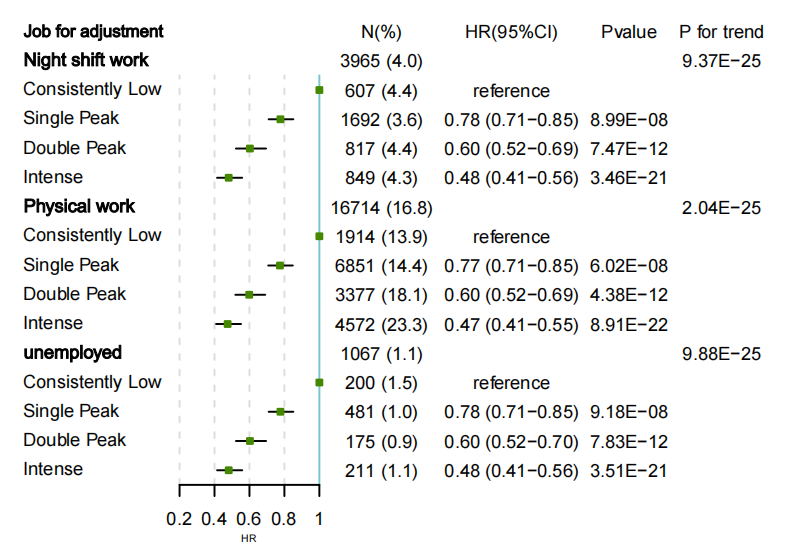


**Figure S6.** The Study flow chart.

**
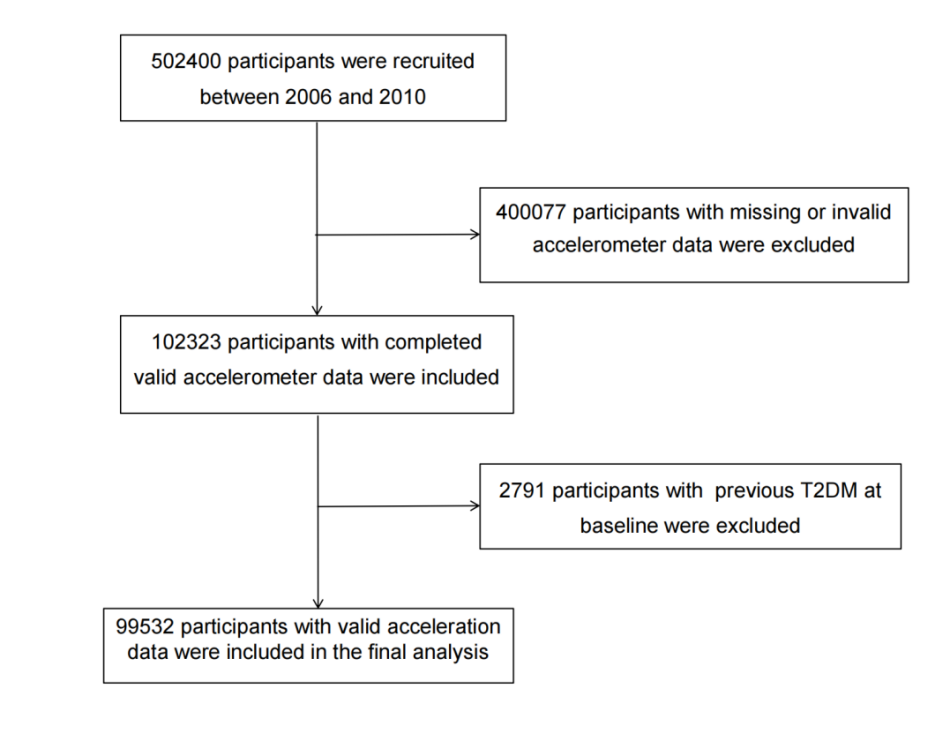
**

**Figure S7.** The Euclidean norm minus one (ENMO) defined as the Euclidean norm for the three-dimensional acceleration for each time point with one gravitational unit being subtracted and negative values truncated to zero:


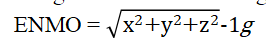


In the UKB, ENMOs were measured in milli gravity (mg) units. Hence, for each participant was measured over the 7-day period. as described by van Hees et al. [1].

1. van Hees VT, Gorzelniak L, Dean León EC, Eder M, Pias M, Taherian S, et al. Separating Movement and Gravity Components in an Acceleration Signal and Implications for the Assessment of Human Daily Physical Activity. PLOS ONE. 2013;8(4):e61691.

**Figure S8.** Within Sum of Square plot. Within Sum of Squares plot to determine number of clusters for K-means clustering analysis. Finally, the number of clusters considered for clustering analysis was four (elbow point).


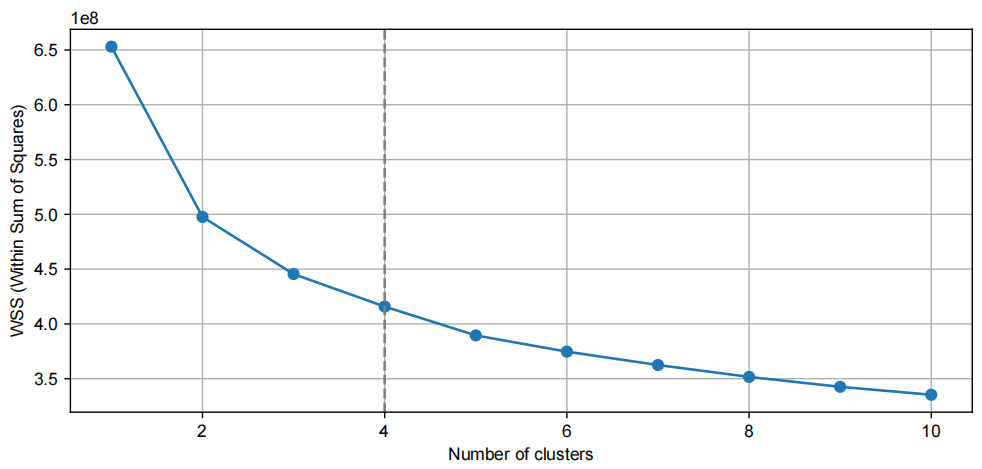


**Figure S9.** Proportional hazard assumptions of PA trajectory patterns and T2DM risk checked by Schoenfeld residuals. Schoenfeld residuals do not vary over time.


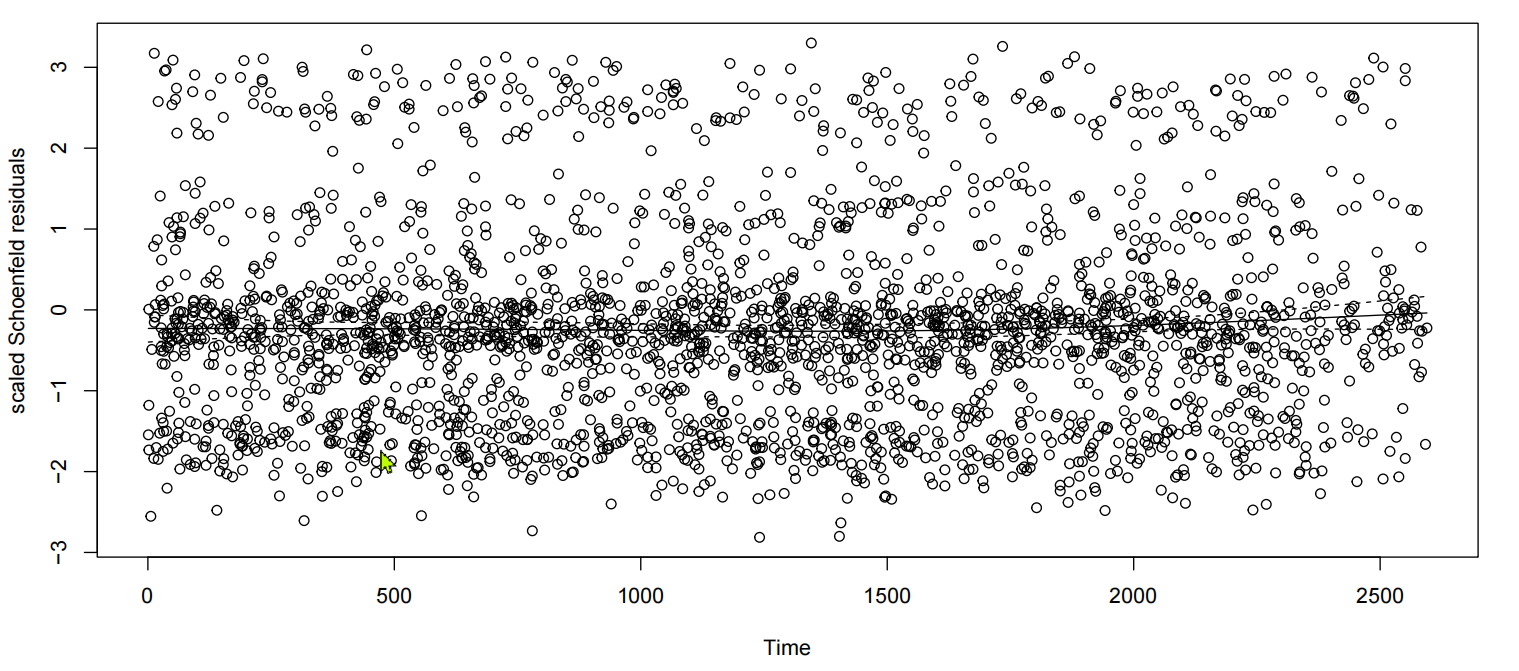


1. Supplementary Tables

| **Time of day** | **mod1** | | | | **mod2** | | | | **mod3** | | | |
| --- | --- | --- | --- | --- | --- | --- | --- | --- | --- | --- | --- | --- |
|  | **HR** | **Lower_95%CI** | **Upper_95%CI** | **P_value** | **HR** | **Lower_95%CI** | **Upper_95%CI** | **P_value** | **HR** | **Lower_95%CI** | **Upper_95%CI** | **P_value** |
| 1 | 1.1 | 1.05 | 1.15 | 1.26E-04 | 1.06 | 1.01 | 1.12 | 1.62E-02 | 1.06 | 1.01 | 1.12 | 2.12E-02 |
| 2 | 1.19 | 1.13 | 1.26 | 5.02E-10 | 1.12 | 1.05 | 1.19 | 5.03E-04 | 1.12 | 1.05 | 1.19 | 7.14E-04 |
| 3 | 1.19 | 1.11 | 1.26 | 1.24E-07 | 1.12 | 1.04 | 1.2 | 4.83E-03 | 1.11 | 1.03 | 1.2 | 6.21E-03 |
| 4 | 1.18 | 1.11 | 1.26 | 9.87E-07 | 1.12 | 1.04 | 1.21 | 3.53E-03 | 1.12 | 1.04 | 1.21 | 4.07E-03 |
| 5 | 1.12 | 1.06 | 1.19 | 1.72E-04 | 1.07 | 0.99 | 1.14 | 8.23E-02 | 1.06 | 0.99 | 1.14 | 9.06E-02 |
| 6 | 1.05 | 1.01 | 1.1 | 1.64E-02 | 1.02 | 0.97 | 1.07 | 4.14E-01 | 1.02 | 0.97 | 1.07 | 3.92E-01 |
| 7 | 0.98 | 0.95 | 1.01 | 1.73E-01 | 0.99 | 0.96 | 1.02 | 5.76E-01 | 0.99 | 0.97 | 1.02 | 5.98E-01 |
| 8 | 0.92 | 0.9 | 0.93 | 2.55E-16 | 0.96 | 0.94 | 0.98 | 2.74E-05 | 0.96 | 0.94 | 0.98 | 4.13E-05 |
| 9 | 0.87 | 0.85 | 0.88 | 7.29E-47 | 0.93 | 0.91 | 0.94 | 2.55E-14 | 0.93 | 0.91 | 0.95 | 5.13E-14 |
| 10 | 0.86 | 0.85 | 0.88 | 6.27E-51 | 0.92 | 0.91 | 0.94 | 9.20E-16 | 0.92 | 0.91 | 0.94 | 1.55E-15 |
| 11 | 0.86 | 0.84 | 0.88 | 4.66E-52 | 0.91 | 0.9 | 0.93 | 6.05E-19 | 0.91 | 0.9 | 0.93 | 1.45E-18 |
| 12 | 0.87 | 0.85 | 0.88 | 1.46E-46 | 0.91 | 0.9 | 0.93 | 2.16E-18 | 0.91 | 0.9 | 0.93 | 2.96E-18 |
| 13 | 0.87 | 0.85 | 0.88 | 7.58E-42 | 0.92 | 0.9 | 0.94 | 1.41E-13 | 0.92 | 0.9 | 0.94 | 2.86E-13 |
| 14 | 0.86 | 0.84 | 0.88 | 8.35E-42 | 0.91 | 0.89 | 0.94 | 2.23E-14 | 0.91 | 0.89 | 0.94 | 3.63E-14 |
| 15 | 0.85 | 0.83 | 0.87 | 1.25E-47 | 0.91 | 0.89 | 0.93 | 1.86E-15 | 0.91 | 0.89 | 0.93 | 2.62E-15 |
| 16 | 0.84 | 0.83 | 0.86 | 8.70E-51 | 0.91 | 0.89 | 0.93 | 1.63E-15 | 0.91 | 0.89 | 0.93 | 1.91E-15 |
| 17 | 0.84 | 0.82 | 0.86 | 4.63E-47 | 0.91 | 0.89 | 0.94 | 1.81E-13 | 0.91 | 0.89 | 0.94 | 1.76E-13 |
| 18 | 0.83 | 0.81 | 0.85 | 1.10E-53 | 0.9 | 0.88 | 0.93 | 1.87E-15 | 0.9 | 0.88 | 0.93 | 2.24E-15 |
| 19 | 0.83 | 0.81 | 0.85 | 2.99E-47 | 0.91 | 0.89 | 0.93 | 1.52E-12 | 0.91 | 0.89 | 0.93 | 1.51E-12 |
| 20 | 0.82 | 0.79 | 0.84 | 2.10E-46 | 0.9 | 0.88 | 0.93 | 4.43E-13 | 0.9 | 0.88 | 0.93 | 5.40E-13 |
| 21 | 0.83 | 0.8 | 0.86 | 5.36E-28 | 0.91 | 0.88 | 0.94 | 2.33E-08 | 0.91 | 0.88 | 0.94 | 2.72E-08 |
| 22 | 0.86 | 0.83 | 0.9 | 7.40E-14 | 0.95 | 0.92 | 0.99 | 1.06E-02 | 0.95 | 0.92 | 0.99 | 1.00E-02 |
| 23 | 0.83 | 0.8 | 0.86 | 1.33E-19 | 0.94 | 0.91 | 0.98 | 4.84E-03 | 0.94 | 0.91 | 0.98 | 4.30E-03 |
| 24 | 0.92 | 0.88 | 0.96 | 7.34E-05 | 0.97 | 0.93 | 1.01 | 1.85E-01 | 0.97 | 0.93 | 1.01 | 1.74E-01 |

**Table S1.** Association of T2DM with 24-hourly acceleration. Model 1 adjusted for gender, age, and ethnicity. Model 2 further adjusted for education level, income, BMI, and smoking based on Model 1. Model 3 further adjusted for eGFR, CRP, and medical history of cardiovascular disease and cancer based on Model 2.

**Table S2.** Association of T2DM with 24-hourly acceleration, further excluding individuals with HbA1c levels≥48 mmol/mol (6.5%) or random glucose levels≥11.1 mmol/L at baseline. Model 1 adjusted for gender, age, and ethnicity. Model 2 further adjusted for education level, income, BMI, and smoking based on Model 1. Model 3 further adjusted for eGFR, CRP, and medical history of cardiovascular disease and cancer based on Model 2.

| **Time of day** | **HR** | **Lower_95%CI** | **Upper_95%CI** | **P_value** | **HR** | **Lower_95%CI** | **Upper_95%CI** | **P_value** | **HR** | **Lower_95%CI** | **Upper_95%CI** | **P_value** |
| --- | --- | --- | --- | --- | --- | --- | --- | --- | --- | --- | --- | --- |
| 1 | 1.09 | 1.03 | 1.15 | 1.53E-03 | 1.06 | 1 | 1.12 | 6.20E-02 | 1.05 | 1 | 1.11 | 7.29E-02 |
| 2 | 1.17 | 1.1 | 1.25 | 1.44E-06 | 1.09 | 1.02 | 1.18 | 1.92E-02 | 1.09 | 1.01 | 1.17 | 2.35E-02 |
| 3 | 1.17 | 1.09 | 1.26 | 2.84E-05 | 1.09 | 1 | 1.19 | 4.73E-02 | 1.09 | 1 | 1.19 | 5.32E-02 |
| 4 | 1.17 | 1.09 | 1.26 | 5.49E-05 | 1.11 | 1.02 | 1.21 | 2.12E-02 | 1.11 | 1.02 | 1.21 | 2.24E-02 |
| 5 | 1.11 | 1.04 | 1.19 | 3.78E-03 | 1.05 | 0.97 | 1.14 | 2.17E-01 | 1.05 | 0.97 | 1.14 | 2.20E-01 |
| 6 | 1.05 | 1 | 1.1 | 5.46E-02 | 1.02 | 0.96 | 1.07 | 5.28E-01 | 1.02 | 0.97 | 1.08 | 4.89E-01 |
| 7 | 0.97 | 0.94 | 1 | 5.70E-02 | 0.98 | 0.95 | 1.01 | 2.55E-01 | 0.98 | 0.95 | 1.01 | 2.89E-01 |
| 8 | 0.91 | 0.89 | 0.93 | 1.42E-14 | 0.95 | 0.93 | 0.97 | 4.13E-05 | 0.95 | 0.93 | 0.98 | 8.09E-05 |
| 9 | 0.87 | 0.85 | 0.88 | 1.90E-38 | 0.93 | 0.91 | 0.95 | 3.56E-12 | 0.93 | 0.91 | 0.95 | 9.73E-12 |
| 10 | 0.86 | 0.85 | 0.88 | 2.24E-41 | 0.92 | 0.9 | 0.94 | 3.55E-13 | 0.92 | 0.9 | 0.94 | 1.04E-12 |
| 11 | 0.86 | 0.85 | 0.88 | 1.43E-38 | 0.92 | 0.9 | 0.94 | 1.46E-13 | 0.92 | 0.9 | 0.94 | 5.66E-13 |
| 12 | 0.87 | 0.85 | 0.89 | 9.45E-36 | 0.92 | 0.9 | 0.94 | 3.78E-14 | 0.92 | 0.9 | 0.94 | 1.02E-13 |
| 13 | 0.87 | 0.85 | 0.89 | 1.27E-32 | 0.92 | 0.9 | 0.95 | 9.68E-11 | 0.93 | 0.9 | 0.95 | 3.21E-10 |
| 14 | 0.86 | 0.84 | 0.88 | 4.25E-31 | 0.92 | 0.9 | 0.94 | 1.43E-10 | 0.92 | 0.9 | 0.95 | 3.63E-10 |
| 15 | 0.85 | 0.83 | 0.88 | 6.44E-37 | 0.92 | 0.89 | 0.94 | 5.10E-12 | 0.92 | 0.9 | 0.94 | 1.39E-11 |
| 16 | 0.85 | 0.83 | 0.87 | 1.95E-37 | 0.92 | 0.9 | 0.94 | 5.09E-11 | 0.92 | 0.9 | 0.94 | 1.19E-10 |
| 17 | 0.85 | 0.83 | 0.87 | 5.60E-36 | 0.92 | 0.9 | 0.94 | 3.01E-10 | 0.92 | 0.9 | 0.94 | 5.71E-10 |
| 18 | 0.83 | 0.81 | 0.85 | 2.22E-41 | 0.91 | 0.88 | 0.93 | 8.45E-12 | 0.91 | 0.88 | 0.93 | 1.90E-11 |
| 19 | 0.83 | 0.81 | 0.86 | 7.59E-36 | 0.92 | 0.89 | 0.94 | 3.09E-09 | 0.92 | 0.89 | 0.94 | 5.10E-09 |
| 20 | 0.82 | 0.79 | 0.84 | 1.45E-36 | 0.9 | 0.88 | 0.93 | 2.27E-10 | 0.9 | 0.88 | 0.93 | 4.20E-10 |
| 21 | 0.84 | 0.81 | 0.87 | 4.13E-21 | 0.92 | 0.88 | 0.95 | 3.48E-06 | 0.92 | 0.89 | 0.95 | 5.66E-06 |
| 22 | 0.87 | 0.83 | 0.91 | 1.03E-10 | 0.96 | 0.92 | 1 | 3.87E-02 | 0.96 | 0.92 | 1 | 4.26E-02 |
| 23 | 0.84 | 0.8 | 0.87 | 1.01E-14 | 0.95 | 0.91 | 0.99 | 2.91E-02 | 0.95 | 0.91 | 0.99 | 2.96E-02 |
| 24 | 0.93 | 0.89 | 0.97 | 1.69E-03 | 0.98 | 0.94 | 1.03 | 4.48E-01 | 0.98 | 0.94 | 1.03 | 4.52E-01 |

**Table S3.** Comparison of participant characteristics in the UK Biobank Study across four physical activity trajectory groups.

| **Trajectory cluster** | **Overall** | Consistently Low | Single Peak | Double Peak | Intense | *p* | | | | | | |
| --- | --- | --- | --- | --- | --- | --- | --- | --- | --- | --- | --- | --- |
| N(%) | 99532 | 13764(13.8%) | 47500(47.7%) | 18681(18.8%) | 19587(19.7%) | overall | Consistently Low vs Single Peak | Consistently Low vs Double Peak | Consistently Low vs Intense | Single Peak vs Double Peak | Single Peak vs Intense | Double Peak vs Intense |
| Men (%) | 42902(43.1) | 7096 (51.6) | 20350 42.8) | 7693 (41.2) | 7763 (39.6) | 5.62E-113 | 1.35E-72 | 4.18E-76 | 2.29E-102 | 1.37E-04 | 3.65E-14 | 2.56E-03 |
| Age | 56.0 (7.8) | 57.3 (7.9) | 57.5 (7.4) | 51.9 (7.6) | 55.3 (7.5) | 1.96E-306 | 2.56E-03 | 1.96E-306 | 7.77E-115 | 1.96E-306 | 9.59E-257 | 1.96E-306 |
| Race and ethnicity (%) |  |  |  |  |  | 4.90E-64 | 8.52E-11 | 6.29E-08 | 1.64E-11 | 1.08E-54 | 1.48E-01 | 7.84E-39 |
| White | 96444 (96.9) | 13263 (96.4) | 46303 (97.5) | 17761 (95.1) | 19117 (97.6) | 2.62E-65 | 3.40E-12 | 3.69E-08 | 5.81E-11 | 7.16E-56 | 3.78E-01 | 2.45E-39 |
| Mixed | 550 (0.6) | 78 (0.6) | 210 (0.4) | 166 (0.9) | 96 (0.5) | 1.14E-10 | 6.79E-02 | 1.19E-03 | 3.57E-01 | 1.10E-11 | 4.16E-01 | 3.44E-06 |
| Asian or Asian British | 1160 (1.2) | 215 (1.6) | 461 (1.0) | 322 (1.7) | 162 (0.8) | 3.83E-22 | 8.19E-09 | 2.78E-01 | 6.91E-10 | 1.44E-15 | 8.76E-02 | 8.45E-15 |
| Black or Black British | 844 (0.8) | 131 (1.0) | 313 (0.7) | 289 (1.5) | 111 (0.6) | 1.11E-31 | 4.86E-04 | 4.07E-06 | 6.33E-05 | 5.83E-27 | 1.85E-01 | 9.32E-21 |
| Other ethnic group | 534 (0.5) | 77 (0.6) | 213 (0.4) | 143 (0.8) | 101 (0.5) | 1.70E-05 | 1.06E-01 | 2.98E-02 | 5.98E-01 | 7.89E-07 | 2.65E-01 | 2.66E-03 |
| Educational level (%) |  |  |  |  |  | 5.18E-100 | 3.02E-03 | 7.80E-66 | 3.76E-01 | 9.38E-79 | 2.21E-02 | 2.36E-73 |
| College or University degree | 43569 (43.8) | 5778 (42.0) | 20446 (43.0) | 9188 (49.2) | 8157 (41.6) | 2.98E-61 | 3.03E-02 | 1.85E-37 | 5.54E-01 | 6.66E-46 | 1.13E-03 | 3.98E-49 |
| A levels/AS levels or equivalent | 13159 (13.2) | 1805 (13.1) | 6170 (13.0) | 2623 (14.0) | 2561 (13.1) | 4.15E-03 | 7.10E-01 | 1.93E-02 | 9.17E-01 | 4.52E-04 | 7.69E-01 | 6.95E-03 |
| O levels/GCSEs or equivalent | 20379 (20.5) | 2746 (20.0) | 9836 (20.7) | 3590 (19.2) | 4207 (21.5) | 2.82E-07 | 6.04E-02 | 1.10E-01 | 9.54E-04 | 2.53E-05 | 2.99E-02 | 6.37E-08 |
| CSEs or equivalent | 4038 (4.1) | 510 (3.7) | 1701 (3.6) | 821 (4.4) | 1006 (5.1) | 2.30E-20 | 5.04E-01 | 2.51E-03 | 1.11E-09 | 1.29E-06 | 2.80E-20 | 9.01E-04 |
| NVQ or HND or HNC or equivalent | 5293 (5.3) | 862 (6.3) | 2578 (5.4) | 820 (4.4) | 1033 (5.3) | 3.55E-12 | 2.42E-04 | 1.04E-13 | 1.68E-04 | 8.11E-08 | 4.37E-01 | 7.78E-05 |
| Other | 13094 (13.2) | 2063 (15.0) | 6769 (14.3) | 1639 (8.8) | 2623 (13.4) | 8.36E-87 | 3.46E-02 | 3.22E-67 | 5.09E-05 | 3.58E-80 | 4.33E-03 | 2.95E-46 |
| Household income %) |  |  |  |  |  | 2.90E-297 | 4.00E-08 | 3.17E-201 | 3.72E-30 | 2.32E-255 | 1.10E-18 | 1.46E-103 |
| Low | 14457 (14.5) | 2588 (18.8) | 7497 (15.8) | 1706 (9.1) | 2666 (13.6) | 1.64E-153 | 8.27E-17 | 1.83E-141 | 3.63E-37 | 6.04E-109 | 1.83E-12 | 1.11E-42 |
| Medium | 53373 (53.6) | 7287 (52.9) | 26211 (55.2) | 9193 (49.2) | 10682 (54.5) | 4.93E-43 | 4.96E-06 | 5.38E-11 | 4.90E-03 | 3.10E-43 | 1.39E-01 | 4.47E-25 |
| High | 31702 (31.9) | 3889 (28.3) | 13792 (29.0) | 7782 (41.7) | 6239 (31.9) | 2.26E-233 | 8.45E-02 | 1.54E-135 | 3.66E-12 | 2.54E-212 | 7.99E-13 | 2.30E-87 |
| BMI, kg/m2 | 26.6 (4.4) | 28.4 (5.2) | 27.0 (4.4) | 25.9 (4.1) | 25.3 (3.6) | 1.96E-306 | 1.42E-201 | 1.96E-306 | 1.96E-306 | 3.58E-207 | 1.96E-306 | 5.66E-52 |
| Smoking status (%) |  |  |  |  |  | 1.88E-80 | 1.86E-33 | 8.93E-74 | 6.12E-49 | 2.52E-25 | 3.41E-08 | 2.25E-05 |
| Never | 57116 (57.4) | 7189 (52.2) | 26964 (56.8) | 11458 (61.3) | 11505 (58.7) | 3.49E-62 | 8.70E-21 | 7.35E-60 | 1.11E-31 | 1.92E-26 | 3.96E-06 | 3.32E-07 |
| Previous | 35445 (35.6) | 5077 (36.9) | 17292 (36.4) | 6076 (32.5) | 7000 (35.7) | 2.67E-21 | 3.19E-01 | 6.02E-16 | 3.64E-02 | 1.19E-20 | 1.13E-01 | 6.11E-11 |
| Current | 6971 (7.0) | 1498 (10.9) | 3244 (6.8) | 1147 (6.1) | 1082 (5.5) | 2.72E-87 | 7.84E-55 | 3.19E-53 | 4.12E-72 | 1.72E-03 | 6.60E-10 | 1.21E-02 |
| eGFR, mL/(min*1.73m^2) | 91.3 (12.6) | 89.6 (13.7) | 90.0 (12.6) | 94.6 (12.1) | 92.6 (11.9) | 1.96E-306 | 2.56E-03 | 2.39E-254 | 4.36E-99 | 1.96E-306 | 4.57E-135 | 1.38E-56 |
| CRP, mg/L | 2.2 (3.9) | 3.0 (4.9) | 2.4 (3.9) | 1.9 (3.5) | 1.8 (3.3) | 1.08E-207 | 2.36E-50 | 2.18E-117 | 4.30E-153 | 1.39E-47 | 3.35E-76 | 1.69E-03 |
| Medical history |  |  |  |  |  |  |  |  |  |  |  |  |
| Cardiovascular disease (%) | 11937 (12.0) | 2307 (16.8) | 6176 (13.0) | 1556 (8.3) | 1898 (9.7) | 9.20E-146 | 6.07E-29 | 5.68E-118 | 4.68E-81 | 4.29E-63 | 1.05E-32 | 4.98E-06 |
| Cancer (%) | 10617 (10.7) | 1610 (11.7) | 5402 (11.4) | 1671 (8.9) | 1934 (9.9) | 1.37E-23 | 3.11E-01 | 8.84E-16 | 1.62E-07 | 1.91E-19 | 2.55E-08 | 2.40E-03 |

**Table S4.** Covariate data missingness and imputation in this study.

|  | **Original Data** | **Imputed Data** | **Missing Percentage (%)** | **Imputed or not** |
| --- | --- | --- | --- | --- |
| N (%) | 99532 | 99532 |  |  |
| Male (%) | 42902 (43.10) | 42902 (43.10) | 0.00 | No |
| Age | 56.0 (7.8) | 56.0 (7.8) | 0.00 | No |
| Race and ethnicity (%) |  |  | 0.35 | Yes |
| White | 96099 (96.89) | 96444 (96.90) |  |  |
| Mixed | 550 (0.55) | 550 (0.55) |  |  |
| Asian or Asian British | 1158 (1.17) | 1160 (1.17) |  |  |
| Black or Black British | 843 (0.85) | 844 (0.85) |  |  |
| Other ethnic group | 534 (0.54) | 534 (0.54) |  |  |
| Educational level (%) |  |  | 0.99 | Yes |
| College or University degree | 43010 (43.65) | 43569 (43.77) |  |  |
| A levels/AS levels or equivalent | 13073 (13.27) | 13159 (13.22) |  |  |
| O levels/GCSEs or equivalent | 20229 (20.53) | 20379 (20.47) |  |  |
| CSEs or equivalent | 3998 (4.06) | 4038 (4.06) |  |  |
| NVQ or HND or HNC or equivalent | 5252 (5.33) | 5293 (5.32) |  |  |
| Other | 12981 (13.17) | 13094 (13.16) |  |  |
| Household income (%) |  |  | 10.31 | Yes |
| Low | 12846 (14.39) | 14457 (14.52) |  |  |
| Medium | 47157 (52.83) | 53373 (53.62) |  |  |
| High | 29266 (32.78) | 31702 (31.85) |  |  |
| BMI, kg/m2 | 26.63 (4.43) | 26.63 (4.43) | 0.21 | Yes |
| Smoking status (%) |  |  | 0.27 | Yes |
| Never | 56954 (57.37) | 57116 (57.38) |  |  |
| Previous | 35361 (35.62) | 35445 (35.61) |  |  |
| Current | 6953 (7.00) | 6971 (7.00) |  |  |
| eGFR, mL/(min*1.73m^2) | 91.32 (12.63) | 91.32 (12.64) | 5.88 | Yes |
| CRP, mg/L | 2.22 (3.91) | 2.23 (3.90) | 6.03 | Yes |
| Medical history |  |  |  |  |
| Cardiovascular disease (%) | 11937 (11.99) | 11937 (11.99) | 0.00 | No |
| Cancer(%) | 10617 (10.67) | 10617 (10.67) | 0.00 | No |
